# A Runtime Analysis of Parallel Evolutionary Algorithms in Dynamic Optimization

**DOI:** 10.1007/s00453-016-0262-4

**Published:** 2016-12-07

**Authors:** Andrei Lissovoi, Carsten Witt

**Affiliations:** 1 Department of Computer Science, University of Sheffield, Sheffield, UK; 2 DTU Compute, Technical University of Denmark, Kongens Lyngby, Denmark

**Keywords:** Evolutionary algorithms, Island models, Dynamic problems, Populations, Runtime analysis

## Abstract

A simple island model with $\lambda$ islands and migration occurring after every $\tau$ iterations is studied on the dynamic fitness function Maze. This model is equivalent to a $(1+\lambda )$ EA if $\tau =1$, i. e., migration occurs during every iteration. It is proved that even for an increased offspring population size up to $\lambda =O(n^{1-\epsilon })$, the $(1+\lambda )$ EA is still not able to track the optimum of Maze. If the migration interval is chosen carefully, the algorithm is able to track the optimum even for logarithmic $\lambda$. The relationship of $\tau , \lambda$, and the ability of the island model to track the optimum is then investigated more closely. Finally, experiments are performed to supplement the asymptotic results, and investigate the impact of the migration topology.

## Introduction

Evolutionary algorithms (EAs) are a class of nature-inspired algorithms which can be applied to solve a wide variety of optimization problems. Rigorous runtime analysis of nature-inspired algorithms, building on mathematical methods from the analysis of classical algorithms, has advanced considerably in recent years [3, 22]. While most of these studies focus on so-called static optimization problems, whose set of optima is fixed, there has been increasing interest in the analysis of evolutionary and other nature-inspired algorithms on so-called dynamic problems. Many real-world optimization problems are subject to dynamics in that the optimal solution may change as the problem conditions change over time, and the algorithms therefore need to be able to not only find or approximate the optimum at some point of time, but also to *track* the optimal solution over time as the problem changes.

Application of EAs to dynamic optimization problems is the subject of study in the Evolutionary Dynamic Optimization field, which in recent years has attracted much activity. Many applications of evolutionary algorithms on dynamic problems are considered in the literature [2, 23], and there are already a number of runtime analyses of evolutionary algorithms for dynamic problems [4, 8, 10, 11, 13, 25]. In particular, the proper use of mechanisms to maintain diversity can be crucial in dynamic optimization [24].

Despite the increasing interest in the area, it has not been well understood what mechanisms allow EAs or related nature-inspired algorithms to efficiently track the optimum of a dynamic problem. In [14], Kötzing and Molter introduced a dynamic pseudo-boolean function called Maze that separates simple evolutionary algorithms and ant colony optimization. More precisely, the paper shows that while a Max-Min Ant System (MMAS) is able to track the changes occurring in the Maze fitness function and finds the optimum within polynomial time, a (1+1) EA loses track of the optimum and requires with high probability an exponential amount of time to find the optimum. Very recently, we have built upon this study [19], extending the Maze function to finite alphabets, and proving that with an appropriately-chosen ancestor population size $\mu$, a $(\mu +1)$ evolutionary algorithm is able to track the optimum when a genotype diversity mechanism is used. Additionally, a simple MMAS is proved insensitive with respect to the parameter as it is able to track the optimum without any modifications for a wide range of values for $\mu$.

In this work, we consider a different mechanism and analyze its behavior and limitations in tracking the optimum of the Maze benchmark function. We focus on parallel nature-inspired algorithms, which are heavily employed in practice due the rapid development of parallel computer architectures. The survey by Alba, Luque and Nesmachnow [1] describes important applications and theoretical studies in this area. In particular, it refers to experiments with parallel nature-inspired algorithms in dynamic optimization, including a study of a parallel swarm algorithm for dynamic vehicle routing problems [12]. It is therefore interesting to determine the theoretical properties of parallel nature-inspired algorithms that allow them to track the optimum of a dynamic problem, or cause them to fail to do so. Both the number of so-called islands (independent subpopulations) and the communication between them seem influential. From a more general perspective, [23] emphasizes the usefulness of memory and diversity-maintaining operators in EAs for dynamic optimization.

In theoretical runtime analysis, there is already a body on results on parallel evolutionary algorithms. This field was pioneered by Lässig and Sudholt [15], who proved that the proper use of island models with a dense topology and regular migration can reduce the runtime by exponential factors compared to independent EAs. They also give examples where improper choices of parameters such as the migration intervals can lead to poor performance of the island model and investigate the role of sparse communication topologies. Moreover, general results on the speedup achievable through different topologies are available [16]. It is also known that island models can support the effect of crossover [21] and yield exponential speedups on specific examples. Still, to the best of our knowledge, runtime analyses of parallel EAs on dynamic optimization problems have not been available so far.

Our contribution is represented by a runtime analysis of a parallel EA for the dynamic Maze problem. We define a simple parallel EA using an island model with communication occurring within regular intervals, the above-mentioned migration intervals, in the style of [15]. The impact of two parameters is studied, namely the number of islands $\lambda$ and length of the migration intervals $\tau$. We observe that the choice of migration interval has a similar effect to what is proved in [15] for static optimization, though with respect to a completely different function. In a nutshell, our results are as follows. In the extreme case that $\tau =1$, i. e., migration occurs in every generation, the model boils down to a $(1+\lambda )$ EA. It is shown that offspring population sizes, i. e., number of islands, of up to $\lambda =O(n^{1-\epsilon })$, where *n* is the problem size and $\epsilon$ an arbitrarily small positive constant, do not allow this algorithm to track the Maze efficiently. In contrast, if $\tau$ is chosen appropriately, ensuring that migration does not occur too frequently, already $\lambda ={\varOmega }(\log n)$ islands allow efficient tracking of the optimum of the Maze. Moreover, more general conditions on the choice of $\tau$ are worked out, resulting in either efficient tracking or, if the migration schedule is not chosen carefully, losing track of the optimum. To the best of our knowledge, our contribution represents the first runtime analysis of parallel EAs in dynamic optimization. The results indicate that carefully choosing the migration policy and thereby the communication strategy of an island model can be more advantageous than a mere increase of offspring population size.

This paper is structured as follows. In Sect. 2, we introduce the parallel EA and the dynamic optimization problem Maze studied throughout the paper, and define important tools used in the analysis. Section 3 is concerned with the negative result for the parallel EA with $\tau =1$, i. e., the $(1+\lambda )$ EA. The case of appropriately chosen $\tau$, leading to efficient tracking with a small number of islands, is analyzed in Sect. 4. Moreover, the section elaborates on the impact of the choice of $\tau$ on efficient tracking in a more general sense, demonstrating that the approach loses track of the optimum if the parameters of the island model and migration policy are chosen in an unfortunate manner. Section 5 validates the theoretical results of previous sections by presenting experimental results. We finish with some conclusions.

## Preliminaries

The Maze fitness function, proposed in [14], and defined formally below, consists of $n+1$ phases of $t_0 = k n^3 \log n$ iterations each. This phase length was used in [14] to allow the Max-Min Ant System (MMAS) algorithm time to adjust the pheromone values during each phase, and is preserved here for mostly historical reasons. For convenience, we will assume that *k* is chosen such that $t_0$ is a multiple of 3.

During the first phase of the Maze, which we will for convenience refer to as phase 0, the function is equivalent to OneMax: the fitness of an *n*-bit string is equal to the number of 1-bits in the string. In the next *n* phases, higher fitness values $n+1$ and $n+2$ are assigned to two bit strings determined by the phase in an oscillating pattern: every two iterations out of three, $\hbox {OPT}_p = 0^p1^{n-p}$ is assigned the fitness value $n+2$, while $\hbox {ALT}_p = 0^{p-1}1^{n-p+1}$ is assigned the fitness value $n+1$, and during every third iteration, these assignments are reversed; all other bit strings retain their OneMax values. Notably, for $0 \le p < n$, $\hbox {OPT}_p$ is equal to $\hbox {ALT}_{p+1}$. Past the last oscillating phase (“phase *n*”), Maze behaves in a fashion similar to Trap: all individuals except $0^n$ are assigned OneMax values, while $0^n$ is the global optimum, being assigned the highest fitness value. [14] proves that a (1+1) EA loses track of the optimum of this Maze function, reverting to optimizing OneMax, and is therefore not able to construct the final $\hbox {OPT}_n = 0^n$ optimum in a polynomial number of iterations.

$$\begin{aligned} \textsc {Maze}(x, t)&= \left\{ \begin{array}{ll} n + 2 &{} \text {if } t> (n+1) \cdot t_0 \,\wedge \, x = 0^n \\ n + 2 &{} \text {if } t> t_0 \,\wedge \, x = \text {OPT}(t) \\ n + 1 &{} \text {if } t > t_0 \,\wedge \, x = \text {ALT}(t) \\ \textsc {OneMax}(x) &{} \text {otherwise} \end{array} \right. \\ \text {OPT}(t)&= \left\{ \begin{array}{ll} \text {OPT}_{\lfloor t / t_0 \rfloor } &{} \text {if }t \ne 0 \mod 3 \\ \text {ALT}_{\lfloor t / t_0 \rfloor } &{} \text {otherwise} \end{array} \right. \\ \text {ALT}(t)&= \left\{ \begin{array}{ll} \text {ALT}_{\lfloor t / t_0 \rfloor } &{} \text {if }t \ne 0 \mod 3 \\ \text {OPT}_{\lfloor t / t_0 \rfloor } &{} \text {otherwise} \end{array} \right. \\ \text {OPT}_p&= 0^p1^{n-p} \quad \text {for } p \le n \\ \text {ALT}_p&= 0^{p-1}1^{n-p+1} \quad \text {for } p \le n \\ \text {ALL}_p&= \{\text {ALT}_p, \text {OPT}_p\} \end{aligned}$$

The fitness difference between the oscillating optimum (and the eventual global optimum at $0^n$) and the OneMax optimum (at $1^n$) could be made arbitrarily large without affecting the presented results. Our results do depend on the presence of the oscillation between OPT and ALT optima within a phase, as well as the equivalence of the $\hbox {OPT}_p$ and $\hbox {ALT}_{p+1}$ individuals. While the Maze is an artificial construction, similar effects may occur in real-world problems: oscillation can be thought to model noisy fitness functions providing uncertain information about which of two good solutions is better, and phase progression might model gradually changing environment conditions.

The clock *t* is considered external to the Maze function, allowing the fitness value of multiple solutions to be evaluated in each clock value *t*. For the $(1+\lambda )$ EA, and the $\lambda$ island model, this corresponds to having hardware available to evaluate many solutions in parallel, or having the problem changes occur at fixed intervals regardless of the number of parallel evaluations.

We consider the behavior of the $(1+\lambda )$ EA, shown as Algorithm 1, and that of a simple island model running $\lambda$ (1+1) EA s in parallel with various choices for the frequency of migration, shown as Algorithm 2, on the Maze function. Both algorithms use the standard bit mutation operator, formalized in Definition 1.

For our purposes, Algorithm 2, if migration is set to occur in every iteration, behaves equivalently to the $(1+\lambda )$ EA on Maze: the algorithms differ only in their initialization of $x^*$ and the first offspring population, which is insignificant as both are able to find the OneMax optimum within the initial phase with high probability. The order of mutation and migration in Algorithm 2 has been selected to allow for this similarity, essentially allowing an ALT individual constructed during an ALT-favoring iteration of the Maze to migrate to all islands, similar to how it would be assigned to $x^*$ in the $(1+\lambda )$ EA.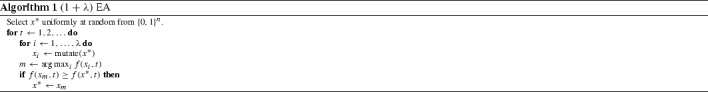 
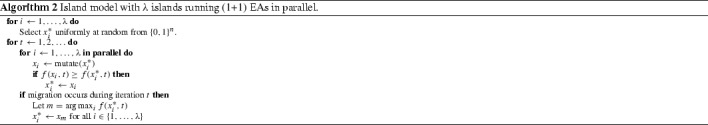


### Definition 1

(*Standard Bit Mutation*) The mutation operator $\text {mutate}(x)$ creates an image $y \in \{0, 1\}^n$ from $x \in \{0, 1\}^n$ by independently replacing each bit $x_i$ of *x* ($1 \le i \le n$) with $1-x_i$ with probability 1 / *n*.

In the analysis of the $(1+\lambda )$ EA and the simple island model, we make use of Markov chain mixing times to bound the probability that the algorithm is in a particular state (i. e., has a particular individual as $x^*$) after a certain number of iterations. This has been applied to ant colony optimization in [26] and to analyze the $(\mu +1)$ EA on Maze in [19]; for completeness, we repeat the definitions of mixing and coupling times below, closely following the presentation from [26].

### Definition 2

(*Mixing Time*) Consider an ergodic Markov chain over a state space ${\varOmega }$ with stationary distribution $\pi$. Let $p_x^{(t)}$ denote the distribution of the Markov chain *t* iterations after starting in state *x*, and let

$$\begin{aligned} t(\varepsilon ) := \max _{x \in {\varOmega }} \min \left\{ t : \frac{1}{2}\sum _{y \in {\varOmega }} |p_x^{(t)}(y) - \pi (y)| \le \varepsilon \right\} . \end{aligned}$$

The mixing time $t_\text {mix}$ of the Markov chain is then defined as $t_\text {mix} = t(1/(2e))$.

### Definition 3

(*Coupling Time*) Consider a pair process $\left( X^{(t)}, Y^{(t)}\right)$, where both $X^{(t)}$ and $Y^{(t)}$, viewed in isolation, are instances of the same Markov chain. Coupling time $T_{xy}$ is the random time until the two processes, initialized in states *x* and *y* respectively, are in the same state for the first time:

$$\begin{aligned} T_{xy} = \min \{t : X^{(t)} = Y^{(t)} \mid X^{(0)} = x,\, Y^{(0)} = y\}. \end{aligned}$$

The following theorem will be used to bound the mixing time, once a suitable coupling has been established.

### Theorem 4

(Relation Between Mixing and Coupling Time) The worst-case coupling time is an upper bound on the mixing time:

$$\begin{aligned} t(\varepsilon ) \le \min \left\{ t: \max _{x, y \in {\varOmega }} P(T_{xy} > t) \le \varepsilon \right\} . \end{aligned}$$

Additionally, the following multiplicative drift theorem is useful when considering longer migration intervals. It is taken from [6], except for the final tail bound “$P(T > \cdots )<\cdots$”, which stems from [5].

### Theorem 5

(Multiplicative Drift) Let $S \subseteq \mathbb {R}$ be a finite set of positive numbers with minimum $s_{\min } > 0$. Let $\{X^{(t)}\}_{t \ge 0}$ be a sequence of random variables over $S \cup \{0\}$. Let *T* be the random first point in time $t \ge 0$ for which $X^{(t)} = 0$.

Suppose there exists a $\delta > 0$ such that

$$\begin{aligned} E(X^{(t)} - X^{(t+1)} \mid X^{(t)} = s) \ge \delta s \end{aligned}$$

for all $s \in S$ with $P(X^{(t)} = s) > 0$. Then for all $s_0 \in S$ with $P(X^{(0)} = s_0) > 0$,

$$\begin{aligned} E(T \mid X^{(0)} = s_0) \le \frac{\ln (s_0/s_{\min })+1}{\delta } . \end{aligned}$$

Moreover, it holds that $P(T > (\ln (s_0/s_{\min }) + r)/\delta ) \le {\text {e}}^{-r}$ for any $r > 0$.

Several lemmas throughout this paper state that “a specific event occurs with high probability.” Definition 6 provides a more formal definition of this concept.

### Definition 6

An event *E* is said to occur *with high probability* (with respect to the problem size *n*) if, for every constant $c > 0$, $P(E)=1 - O(n^{-c})$.

As a matter of notation, we typically use the variables *c*, $c_1$, etc., to reference local positive constants which could be removed in asymptotic notation.

In general, we say that an algorithm is able to track the optimum of the Maze when it is able to construct the $\hbox {OPT}_n$ individual in polynomial time (with high probability). Typically, this would correspond to maintaining at most a constant Hamming distance to the $\hbox {OPT}_p$ intermediate optima during the oscillating phases.

## The $(1+\lambda )$ EA on Maze

In this section, we analyze the behavior of the $(1+\lambda )$ EA on Maze. As long as $\lambda$ is not too large (trivializing the problem by exploring the entire neighborhood of Hamming distance 1 during every iteration), the EA is not able to track the optimum of the Maze, and reverts to optimizing OneMax. This is formalized in the following theorem, whose proof is inspired by the strategy taken in [19].

### Theorem 7

The $(1+\lambda )$ EA with $\lambda \in O(n^{1-\epsilon })$, for any constant $\epsilon > 0$, will with high probability lose track of the optimum of Maze, i. e., with high probability it will require an exponential number of iterations to construct $\hbox {OPT}_n$.

We first note that the EA is able to find $\text {OPT}_0 = 1^n$ successfully, but then has at least a constant probability of ending each of the following *n* oscillating phases with $x^* \ne \text {OPT}_p$, and at least a constant probability of ending the next phase after at least a constant fraction of such phases with $x^* = 1^n$; if this occurs sufficiently late in the optimization process, constructing an $\hbox {ALL}_p$ individual from $1^n$ requires a large simultaneous mutation, the waiting time for which is exponential with respect to *n*.

The following lemma already follows from a more general result in [7], which states that the $(1+\lambda )$ EA optimizes all linear functions in $O((n\log n)/\lambda + n)=O(n\log n)$ generations with high probability. Moreover, [9] shows the bound $((n\log n)/\lambda + n\log \lambda /(\log \log \lambda ))=O(n\log n)$ for the special case of the OneMax function, which we consider here. For the sake of completeness, we give a simple proof of the $O(n\log n)$ bound for this special case.

### Lemma 8

The $(1+\lambda )$ EA constructs $\hbox {OPT}_0 = 1^n$ during phase 0 of the Maze, which consists of $t_0 = O(n^3 \log n)$ iterations, with high probability.

### Proof

Phase 0 of the Maze consists of $t_0 = k n^3 \log n$ iterations during which the Maze fitness function is equivalent to OneMax. It is left to show that the $(1+\lambda )$ EA constructs the OneMax optimum in $O(n \log n)$ iterations with high probability (where the implicit constant in the *O*-notation would depend on the constant *c* chosen in Definition 6); notably, $t_0$ is asymptotically larger than $n \log n$. Our simple proof uses well-known arguments that are also valid for the case $\lambda =1$, i. e., the $(1+1)$ EA. We note that while increasing $\lambda$ may decrease the number of expected generations to optimize OneMax, it does not decrease the expected number of function evaluations, see [9].

To prove the claim that the $(1+\lambda )$ EA constructs the OneMax optimum in $O(n \log n)$ iterations with high probability, we apply Theorem 5, defining $X^{(t)}$ as the number of zero-bits in the bit string $x^*$ during iteration *t*, and considering the drift:

$$\begin{aligned} E(X^{(t)} - X^{(t+1)} \mid X^{(t)} = x)&\ge \frac{x}{n}(1-1/n)^{n-1} \ge \frac{1}{ne} x. \end{aligned}$$

This lower bound is obtained by analyzing the event that the first offspring flips a zero-bit and does not flip any one-bits, which is sufficient to decrease the number of zero-bits by at least 1 towards the next iteration.

As at most *n* bits are wrong initially, the expected optimization time *T* is:

$$\begin{aligned} E(T \mid X^{(0)} \le n)&\le \frac{\ln (n)+1}{1/(ne)} = ne \ln (n) + ne . \end{aligned}$$

Applying the tail-bound with $r = c_1 \ln n$ yields an upper bound on the probability of exceeding the expected number of iterations to reach $X^{t} = 0$ (i. e., to find the $1^n$ optimum) by more than $c_2 n \ln n$ additional iterations, where both $c_1, c_2$ are constants:

$$\begin{aligned} {\text {e}}^{-c_1 \ln n} = n^{-c_1} , \end{aligned}$$

i. e., $1^n$ is constructed by the $(1+\lambda )$ EA in $ne \ln (n) + ne + c_2 n \ln n = O(n \log n)$ iterations with probability $1 - n^{-c_1}$. $\square$

### Lemma 9

Given that $x^* \in \text {ALL}_p$ at least $c n/\lambda$ iterations before the end of phase *i*, and $\lambda \in o(n)$, the probability that phase *i* ends with $x^* = \text {OPT}_i$ is in ${\varTheta }(1)$, and the probability that phase *i* ends with $x^* = \text {ALT}_p$ is also in ${\varTheta }(1)$.

### Proof

Once an $\hbox {ALL}_i$ individual is constructed, only mutations which construct $\hbox {OPT}_i$ or $\hbox {ALT}_i$ can be accepted during the remainder of the phase, with single-bit mutations at specific iterations of the oscillation allowing the EA to switch between the two.

Let $p_1$ be the probability of a specific single-bit mutation occurring in a single iteration of the $(1+\lambda )$ EA, i. e., the probability that $\hbox {OPT}_i$ is constructed from $\hbox {ALT}_i$ or vice versa:

$$\begin{aligned} p_1 = 1 - \left( 1 - \left( 1 - 1/n\right) ^{n-1} \cdot 1/n\right) ^\lambda = {\varTheta }(\lambda /n), \end{aligned}$$

noting that $\lambda /(2en) \le p_1 \le \lambda /(2n)$ for $n \ge 2$.

Consider the phase as a series of oscillations, each oscillation consisting of three iterations. During the first two iterations of an oscillation, $\hbox {OPT}_n$ has a higher fitness value than $\hbox {ALT}_n$ (and vice versa during the third and final iteration). Let $p_O$ be the probability that the current individual switches from $\hbox {ALT}_i$ to $\hbox {OPT}_i$ during an oscillation; in order for this to occur, $\hbox {OPT}_i$ has to be constructed via a specific single-bit mutation of $\hbox {ALT}_i$ during either the first or the second iteration, and $\hbox {ALT}_i$ must not be constructed via a specific single-bit mutation of $\hbox {OPT}_i$ during the third iteration of the oscillation. Similarly, let $p_A$ be the probability that the current individual switches from $\hbox {OPT}_i$ to $\hbox {ALT}_i$ during an oscillation; in order for this to occur, ALT$_i$ must be constructed from $\hbox {OPT}_i$ via a specific single-bit mutation during the third iteration of the oscillation. Recalling that $p_1$ is the probability of a specific single-bit mutation occurring at least once among $\lambda$ offspring, these probabilities are:

$$\begin{aligned} p_O&= (p_1 + (1-p_1) p_1) (1 - p_1) \\&= (2 - 3 p_1 + {p_1}^2) p_1 = {\varTheta }(\lambda /n) \\ p_A&= p_1 = {\varTheta }(\lambda /n) , \end{aligned}$$

observing that as $p_1 = {\varTheta }(\lambda /n) = o(1)$ as $\lambda = o(n)$, the $2p_1$ term dominates when bounding $p_O$.

The identity of the current individual $x^*$ of the $(1+\lambda )$ EA, observed at the end of each OPT-OPT-ALT oscillation can be modeled using a two-state Markov chain, with one state corresponding to $x^* = \text {OPT}_i$, the other to $x^* = \text {ALT}_i$, and transition probabilities between the states as above.

Let $\pi _O$ and $\pi _A = 1 - \pi _O$ be the steady-state probabilities of $x^* = \text {OPT}_i$ and $x^* = \text {ALT}_i$ respectively; per the definition of a steady-state probability:

$$\begin{aligned} \pi _O p_A&= \pi _A p_O \\ \pi _O p_1&= (1 - \pi _O) p_1 (2 - 3 p_1 + {p_1}^2) \\ \pi _O&= \frac{2 - 3 p_1 + {p_1}^2}{3 - 3 p_1 + {p_1}^2} , \end{aligned}$$

i. e., $\pi _O$ approaches a constant; we note that $\pi _O \le 2/3$, and as $\lambda \in o(n)$ and hence $p_1 \le \lambda /(2n) \le 0.5$, $\pi _O \ge 3/7$.

Over time, the probability of $\hbox {OPT}_i$ being the current individual at the end of an oscillation will approach the steady-state probability $\pi _O$. The number of oscillations requires until this probability is within an $\epsilon$ of $\pi _O$ can be upper-bounded by the mixing time of the Markov chain, which (by Lemma 4) in turn can be upper-bounded by its coupling time, i. e., the maximum number of steps $T_{AO}$ until the probability that two independent instances of the chain initialized in different states are in the same state becomes sufficiently small:

$$\begin{aligned} P(T_{AO} > t)&= (p_Ap_O + (1-p_A)(1-p_O))^t \\&= (1 + 2p_Ap_O - p_A - p_O)^t\\&= (1 - p_1 (3 - 7 p_1 + 7 {p_1}^2 - 2 {p_1}^3))^t \\&\le \left( 1 - \frac{\lambda }{2en}\left( 3 - \frac{7\lambda }{2n} + \frac{7\lambda ^2}{4{\text {e}}^2n^2} - \frac{2\lambda ^3}{8n^3} \right) \right) ^t \\&< \left( 1 - \frac{\lambda }{2en} \right) ^t , \end{aligned}$$

by recalling that $\lambda = o(n)$, and observing that the expression in the inner parentheses is greater than 1 when $\lambda /n \le 0.5$. Then, an upper bound on the coupling time is:

$$\begin{aligned} t(\varepsilon )&\le \min \left\{ t : (1-\lambda /(2en))^t \le \varepsilon \right\} . \end{aligned}$$

After at most $t(0.01) < 9.22en/\lambda$ steps of the Markov chain, i. e., at most $76n/\lambda$ iterations of the $(1+\lambda )$ EA, the probability that $x^* = \text {OPT}_i$ is therefore within $[\pi _O-0.01, \pi _O+0.01]$, and, as $3/7 \le \pi _O \le 2/3$, in ${\varTheta }(1)$. Similarly, the probability that $x^* = \text {ALT}_i$ is within $[1-\pi _O-0.01, 1-\pi _O+0.01]$, and therefore in ${\varTheta }(1)$. $\square$

### Lemma 10

If a phase $i > n/2 + 3$ begins with $x^* \not \in \text {ALL}_{p}$ satisfying $f(x^*) > n - p + 1$, the $(1+\lambda )$ EA with offspring population size $\lambda = O(n^{1-\epsilon })$, for any constant $\epsilon > 0$, ends the phase with $x^* = 1^n$ with at least constant probability.

### Proof

At the start of phase *i*, $x^*$ contains strictly more 1-bits than any individual in $\hbox {ALL}_i$, and the Hamming distance between $x^*$ and the closest $\hbox {ALL}_i$ individual is at least 1. Let $p_R \le p_1$ be the probability that an $\hbox {ALL}_i$ individual is constructed during an iteration.

We want to consider the probability that the number of 1-bits in $x^*$ exceeds that in any $\hbox {ALL}_i$ individual by at least 3 before an $\hbox {ALL}_i$ individual is constructed. An individual with a greater OneMax value is constructed via a single-bit mutation with probability at least $p_{L}$:

$$\begin{aligned} p_{L}&\ge 1 - (1 - n/2 \cdot (1-1/n)^{n-1}/n)^{\lambda } \\&\ge 1 - 0.75^\lambda \ge 1/4 , \end{aligned}$$

as there are at least $n/2\,0$-bits that can be flipped to increase OneMax value. We note that after at most 2 OneMax-improvements, constructing the closest $\hbox {ALL}_i$ individual requires at least 3 1-bits to be flipped simultaneously.

Consider the probability that two OneMax improvements occur before an $\hbox {ALL}_i$ individual is constructed. Let *V* be the event that a OneMax-improving single-bit mutation occurs, and *A* be the event that an $\hbox {ALL}_i$ individual is constructed:

$$\begin{aligned} P(A \mid A \vee V)&\le \frac{p_{R}}{p_{R} + p_{L}} \le \frac{\lambda }{2n \left( \frac{\lambda }{2en} + 1/4\right) } \\&= \frac{2e\lambda }{en + 2\lambda } \in O(\lambda /n), \end{aligned}$$

then, the probability that an $\hbox {ALL}_i$ individual is not constructed before two OneMax-improving single-bit mutations occur is:

$$\begin{aligned} P(2\; V\text {s without }A)&\ge \left( 1 - P(A \mid A \vee V)\right) ^2 \in {\varOmega }(1) . \end{aligned}$$

Once this occurs, constructing an $\hbox {ALL}_i$ individual requires at least 3 specific bits to mutate simultaneously, which with high probability does not happen within the time required to find $1^n$ per Lemma 8. Thus, the $(1+\lambda )$ EA has at least a constant probability of ending the phase with $x^* = 1^n$. $\square$

These lemmas can then be combined to prove Theorem 7.

### Proof

*(of Theorem* 7 *)* With high probability, $\hbox {OPT}_0 = 1^n$ is found during phase 0 per Lemma 8. At the start of each subsequent phase $p, f(x^*) > n-p$, as only individuals in the ALL sets of the preceding phases can be accepted while decreasing the number of 1-bits in $x^*$, and the minimum OneMax value of any individual in sets $\hbox {ALL}_0$,...,ALL$_{p-1}$ is $n-p+1$. Furthermore, if $x^* \not \in \text {ALL}_p, f(x^*) > n-p+1$, as this excludes $x^* = \text {OPT}_{p-1}$, which had the lowest fitness value of all individuals in the union of the previous ALL sets.

If $x^* \ne 1^n$ at the start of phase $p \ge n/2+3$, the phase has at least a constant probability of ending with $x^* \ne \text {OPT}_p$ per Lemma 9, and hence $x^* \not \in \text {ALL}_{p+1}$.

If phase $p+1$ begins with $x^* \not \in \text {ALL}_{p+1}$, it has at least a constant probability of ending with $x^* = 1^n$ per Lemma 10.

Thus, at least a constant fraction of ${\varOmega }(n)$ phases beyond $n/2+3$ have at least a constant probability of ending with $x^* = 1^n$; i. e., with high probability, at least one of those phases will end with $x^* = 1^n$. Constructing an $\hbox {ALL}_p$ individual from $1^n$ in future phases requires at least ${\varOmega }(n)$ bits to be flipped simultaneously, which with high probability does not occur in polynomial time. $\square$

We note that the proof of Theorem 7 relies on $\lambda = o(n)$ primarily in the bounds on $p_1$ in Lemma 9, although, if $\lambda$ is increased a little further to ${\varOmega }(n \log n)$, the behavior described by Lemma 10 would also no longer occur, allowing the $(1+\lambda )$ EA to recover from any phase which ends with an ALT$_p$ with high probability.

### Lemma 11

The $(1+\lambda )$ EA with offspring population size $\lambda \ge c_1 n \log n$, where $c_1 > 0$ is a sufficiently large constant, is able to track the optimum of the Maze function, constructing $\text {OPT}_n$ at the end of the Maze with high probability.

### Proof

Assume that each of the *n* oscillating Maze phases ends with $x_* = \text {ALT}_p$. Consider the probability $p_r$ of constructing an ALL$_{p+1}$ individual in the first iteration of the next phase, which can be lower-bounded by the probability that a specific one-bit mutation occurs (flipping the previously oscillating bit to a 0):

$$\begin{aligned} p_r&= 1 - (1 - (1 - 1/n)^{n-1}/n)^{c_1 n \log n} \\&\ge 1 - (1 - 1/(ne))^{c_1 n \log n} \\&\ge 1 - {\text {e}}^{- c_2 \log n} = 1 - n^{- c_2} . \end{aligned}$$

We can then use a union bound to lower-bound the probability $p_R$ that all *n* phases construct an $\hbox {ALL}_p$ individual in their first iteration:

$$\begin{aligned} p_R \ge (p_r)^n \ge 1 - n^{1-c_2} . \end{aligned}$$

By picking a sufficiently large constant $c_1$ in $\lambda = c_1 n \log n$, we can ensure that the $(1+\lambda )$ EA constructs an $\hbox {ALL}_p$ individual in the first iteration following every phase transition with high probability. $\square$

## A Simple Island Model

Splitting the $\lambda$ offspring onto $\lambda$ islands, which only compare the fitness values of their $x^*$ individuals periodically (for instance, every $\tau$ iterations, where $\tau > 0$ is the migration interval), allows the resulting island model to track Maze even with a modest $\lambda$. In this section, we consider the effect of various migration schedules on how the island model is able to track the Maze.

### With Appropriate Migration Policy

To begin with, consider an island model where migration occurs on the first iteration of every phase, i. e., every $\tau = t_0$ iterations of the Maze. This ensures that an $\hbox {ALL}_p$ individual migrates to all islands if any of the islands end the preceding phase with $x^* = \text {OPT}_{p-1}$.

#### Theorem 12

An island model with $\lambda = c \log n$ islands, where *c* is a sufficiently large constant, each island running a (1+1) EA, and migration occurring during the first iteration of every phase (i. e., with migration interval $\tau = t_0$) is able to find the $\hbox {OPT}_n$ optimum of the Maze with phase length $t_0 = k n^3 \log n$ in polynomial time with high probability.

#### Proof

We note that individually, the islands behave exactly like a (1+1) EA on Maze, and the effects of migration are limited to selecting the best individual at the start of each phase, and propagating it to all islands. Thus, as long as any island ends phase *p* with $x^* = \text {OPT}_p$, all islands will receive an ALL$_{p+1}$ individual during the first iteration of phase $p+1$.

The initial OneMax optimum, $\hbox {OPT}_0$, is found during phase 0 on each island with high probability. Lemma 9, applied with $\lambda = 1$, states that the probability that an island that begins phase *p* with $x^* \in \text {ALL}_p$ ends the phase with $x^* = \text {OPT}_p$ with at least constant probability; let $p_s = {\varOmega }(1)$ be a lower bound on this probability, and $p_f \le (1 - p_s)^\lambda$ an upper bound on the probability that all $\lambda$ islands end the phase with $x^* \ne \text {OPT}_p$. As long as the latter event does not occur, all islands will receive an ALL$_{p+1}$ individual at the start of the next phase, allowing the argument to be repeated inductively. A union bound can then be used to upper-bound the probability of failing in any of the *n* phases:

$$\begin{aligned} 1 - (1 - p_f)^n \le n p_f&\le n (1 - p_s)^\lambda \le n c_1^{c_2 \log n} \le n^{1 + c_2 , \log c_1} \end{aligned}$$

noting that for any constant $c > 0$, choosing $c_2 \ge -(1 + c)/\log c_1$ (recall that $c_1 \le p_f < 1$, so $\log (c_1)$ is negative) results in $p_f \le n^{-c}$.

Thus, with $\lambda = c_2 \log n$ islands, where $c_2$ is a sufficiently large constant, at least one island ends each phase with $x^* = \text {OPT}_p$ with high probability; this individual is propagated to all other islands at the start of the next phase, allowing $\hbox {OPT}_n$ to be constructed and propagated to all islands at the end of the last phase. $\square$

As the proof of Theorem 12 relies primarily on there being enough time between migration and the phase transition for mixing due to mutation to occur on the islands, migration can be allowed to occur more than once within each phase, as long as the final migration within a phase is still sufficiently far from the phase transition.

#### Corollary 13

With the migration interval $\tau \le t_0, \lambda = c_1\log n$ islands are sufficient to track the optimum of the Maze as long as migration occurs at least once during each phase, and no migration occurs during $c_2n$ iterations preceding any phase transition, where $c_1$ and $c_2$ are sufficiently large constants.

#### Proof

As before, the initial OneMax phase is successful with high probability.

Suppose that the final migration occurs $t \ge c_2 n$ iterations before the end of the phase *p*. As long as at least one island had an $\hbox {ALL}_p$ individual as its current solution, all islands will have an $\hbox {ALL}_p$ individual as their current solution following the migration.

We can then apply Lemma 9 to each island (i. e., with $\lambda = 1$); per the Lemma, the probability of an island ending phase *p* with $x^* = \text {OPT}_p$ is at least a constant greater than 0. Thus, the situation at the end of the phase returns to that considered in Theorem 12: each of $\lambda = {\varOmega }(\log n)$ islands has at least a constant probability of ending phase *p* with $x^* = \text {OPT}_p$.

When $\lambda$ is large enough, it holds with high probability that at least one island ends phase *p* with $x^* = \text {OPT}_p$, and hence at least one island will have an $\hbox {ALL}_{p+1}$ individual as its current solution when migration occurs in the next phase, allowing the argument to be repeated inductively for each of *n* phases. Using a union bound to combine the failure probabilities, we can conclude that the island model is able to track the optimum of the Maze with high probability in this setting. $\square$

Thus, we have shown that $\lambda = c_2\log n$ islands running a (1+1) EA are sufficient to track the optimum of the Maze with varying migration intervals, as long as migration occurs at least $c_1n$ iterations before a phase transition, and any migration occurring within $c_1n$ iterations prior to a phase transition occurs only while the OPT individual has a higher fitness value than the ALT individual. In the following section, we will consider how less-careful choices of migration policy policy can prevent the island model from tracking the optimum of the Maze.

### When the Migration Policy is Not Set Appropriately

We note that if migration is allowed to occur an ALT-favoring iteration during the last ${\varOmega }(n)$ iterations of a phase, and an island has ALT as its current solution at that time (which, per Lemma 9, occurs with at least constant probability for each island if OPT-favoring migration has not occurred during the last ${\varOmega }(n)$ iterations), the ALT individual will migrate to all other islands, and mutation may not reconstruct an OPT individual in time for the next phase transition. An example of this setting is illustrated in Theorem 14.

#### Theorem 14

There exist constants $c_1> 0, c_2 > 0$, such that if migration occurs on an ALT-favoring iteration within $c_1n$ iterations of the end of each phase, and no two migrations occur within $c_2n$ iterations of each other, an island model with $\lambda = O(\log n)$ islands will with high probability fail to track the optimum of the Maze.

#### Proof

We will show that if phase $i > n/2 + 3$ begins with no island having a $x^* \in \text {ALL}_{i}$ as its current solution, with at least constant probability, all islands will return to the OneMax optimum. Additionally, we will show that there exist an expected $n^{{\varOmega }(1)}$ phases which start in this configuration, and hence the islands will return to the OneMax optimum before reaching the Maze global optimum at $0^n$ with high probability.

Consider the final migration within phase *i*, which occurs on an ALT-favoring iteration. As all islands have been unaffected by migration for the preceding $c_2n$ iterations, the distribution of the current solutions of islands still tracking the oscillating optimum is close to the steady-state distribution derived in Lemma 9: with at least constant probability, $x^* \ne \text {OPT}_i$ holds for a given island. Hence, with at least constant probability, all islands will have $x^* \ne \text {OPT}_i$ following the migration.

Before the phase transition occurs, at most $(2/3) c_1 \lambda n$ mutations are performed on OPT-favoring iterations; the probability that none of these construct the OPT individual is at least the probability that none of these are a specific 1-bit mutation, which, with an appropriate choice of $c_1$, can be made sufficiently large:

$$\begin{aligned} (1 - (1 - 1/n)^{n-1}/n)^{2 c_1 \lambda n / 3} \ge (1 - 2/(en))^{n 2 c_1 \lambda / 3} \ge n^{-c'} , \end{aligned}$$

where $c' > 0$ is a constant which can be made arbitrarily small by decreasing $c_1$. We note that $c' \le 1/2$ is sufficient: this results in at least an expected $\sqrt{n}$ phases ending without an $\hbox {OPT}_i$ individual among the islands, and a Chernoff bound can be used to show that with probability $1-{\text {e}}^{-{\varOmega }(\sqrt{n})}$, at least $\sqrt{n}/4$ phases beyond phase $n/2 + 2$ end without an $\hbox {OPT}_i$ individual on any island.

When phase *i* ends with no islands having the $\hbox {OPT}_i$ individual as their current solution, phase $i+1$ starts with each island at least a Hamming distance of 1 from any $\hbox {ALL}_{i+1}$ individual. Following the argument of Lemma 10, with probability at least $(1 - O(\lambda /n))^2 \ge 1 - O(\lambda /n)$, an island is likely to revert to optimizing OneMax and reach $x^* = 1^n$ rather than rediscover the oscillating path of the Maze; the probability that this occurs in all islands is then:

$$\begin{aligned} (1 - O(\lambda /n))^\lambda \ge {\text {e}}^{-{\varTheta }(\lambda ^2/n)} = {\varOmega }(1) \end{aligned}$$

as $$\lambda = O(\log n)$$.

Thus, with probability at least $n^{-c'}$, a phase *i* ends with no island having $x^* = \text {OPT}_i$, and with at least constant probability, if no island has $x^* \in \text {ALL}_{i+1}$ at the start of phase $i + 1$ for $i > n/2 + 2$, all islands return to the OneMax optimum, and do not return to the oscillating path without a mutation simultaneously flipping at least *n* / 2 specific bits. With high probability, there are ${\varOmega }(\sqrt{n})$ phases that end without $\hbox {OPT}_i$ on any island, and thus the probability that the island system is able to track the optimum of the Maze through all *n* phases is at most $2^{-{\varOmega }(\sqrt{n})}$. $\square$

While migration intervals longer than the Maze phase length are viable, $\tau$ should not be set too high, as migration is required to repopulate any islands that lose track of the Maze before *all* islands do so if the optimization process is to be successful. We will show that if $\tau \ge c\, t_0 \log \lambda$, $\lambda = O(\log n)$ islands are no longer sufficient to track the Maze.

#### Theorem 15

For $\tau = c_1 \, t_0 \log \lambda$, where $c_1 > 0$ is a sufficiently large constant, $\lambda = O(\log n)$ islands are not sufficient to track the optimum of the Maze.

#### Proof

Consider an interval of $c\, t_0\log \lambda$ iterations during which no migration will occur, where $c_1 \ge c >0$ is a sufficiently large constant, starting at the beginning of some phase *p*, such that $n/2 + 3< p < n - \log \lambda$. Assume that before the start of phase *p*, none of the $\lambda = c \log n$ islands lost track of the Maze, and thus all islands begin phase *p* with at least a constant probability of having an $\hbox {ALL}_p$ individual per Lemma 9.

Considering each island individually, each of the $\log \lambda$ phases in the interval has at least a constant probability of ending with $x^* \ne \text {OPT}_p$, causing the next phase to have at least a constant probability of ending with $x^* = 1^n$; let $p_L > 0$ be a constant lower bound on the probability of each phase ending with $x^* = 1^n$. Let $X^{(t)}$ be the number of islands with $x^* \ne 1^n$ at the start of phase $p+t$ (and $X^0 = \lambda$); it then holds that:

$$\begin{aligned} E(X^{(t)} - X^{(t+1)} \mid X^{(t)}) \ge p_L X^{(t)}, \end{aligned}$$

and hence also

$$\begin{aligned} E(X^{(t)} \mid X^{(0)}) = (1-p_L)^t X^{(0)} . \end{aligned}$$

Theorem 5 can then be applied: the expected number of phase transitions $T = \min _t \{X^{(t)} = 0\}$ until all islands have lost track of the Maze (i. e., have $x^* = 1^n$) is:

$$\begin{aligned} E(T \mid X^{(0)}) \le \frac{\ln (X^{(0)}) + 1}{1-p_L} = O(\ln \lambda ), \end{aligned}$$

and by setting $r:= c\ln \lambda$ in the theorem, where *c* is a sufficiently large constant, we lower bound the probability of all islands ending the interval with $x^* = 1^n$ by 1 / 2 (if $c_1$ is chosen large enough). As there are ${\varOmega }(n/(\log \lambda )) = {\varOmega }(n/(\log \log n))$ such intervals following phase $p > n/2 + 3$, the probability that the island model does lose track of the Maze in at least one of these intervals is at least $1 - 2^{-{\varOmega }(n/\log \log n)}$. $\square$

## Experiments

To supplement the asymptotic results expressed in Theorems 7 and 12, we have also performed simulations of the island model (Algorithm 2) with $\tau =1$ (migration occurring in every iteration) and $\tau =t_0$ (migration occurring at the beginning of each phase) for $n=75$, $t_0 = n^3$, and various choices of $\lambda$.

**Fig. 1:**
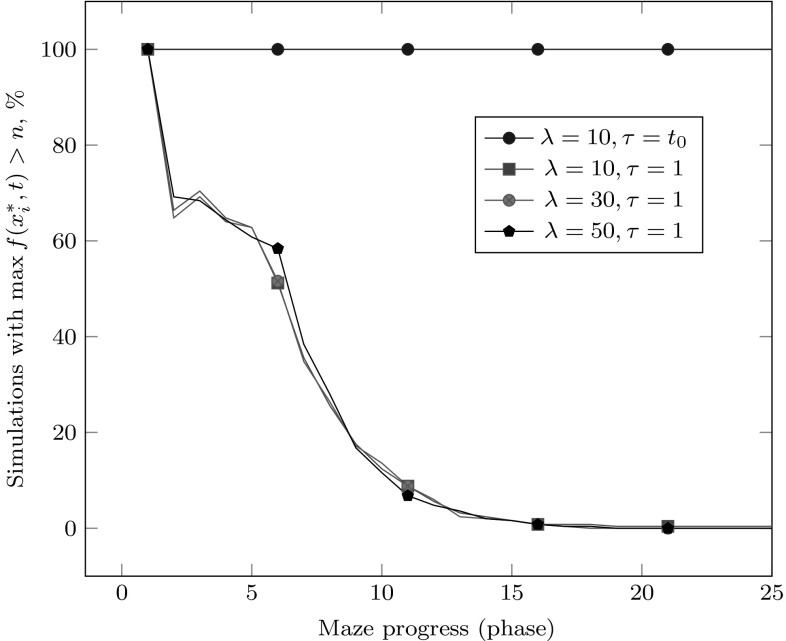
Number of simulations of Algorithm 2 with $n = 75, t_0 = n^3$, and various choices of $\lambda$ and $\tau$, having an individual with a better-than-OneMax value at the start of each Maze phase; 250 simulations for each choice of $\lambda$. Only the first 25 phases are shown here, as there were no further changes observed in the subsequent phases

The results of the simulations are summarized in Fig. 1: $\lambda = 10$ is sufficient for none of the 250 simulations to lose track of the Maze over the $n+1$ phases when $\tau = t_0$, while when $\tau = 1$, all of the simulations performed lose track of the Maze even when $\lambda = 50$. During the first few phases of Maze, it is possible for the islands to reconstruct an $\hbox {ALL}_p$ individual by a modest simultaneous-bit mutation within a single phase, slowing the failure rate of the first few phases; this becomes exponentially less likely as Maze progresses. Notably, increasing $\lambda$ does not have a strong positive effect in the $\tau = 1$ setting for the considered values of $\lambda$, perhaps because each phase ends on an iteration where the ALT individual is assigned a higher fitness value.

**Fig. 2:**
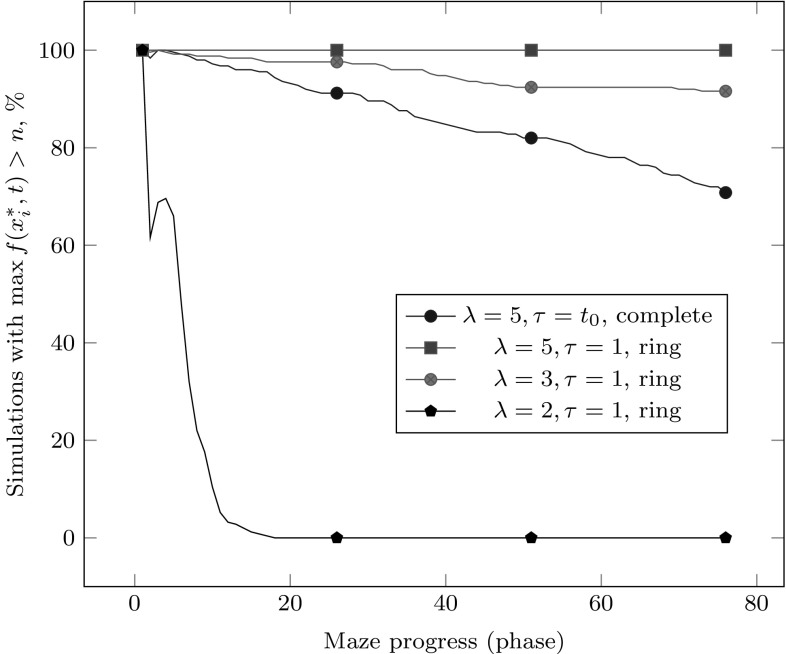
With $n = 75, t_0 = n^3$, with various choices of $\lambda , \tau$, and the migration topology (either a complete graph or a directed ring); 250 simulations in each setting. For $\lambda = 2$, the ring and complete topologies are equivalent

In Algorithm 2, the migration topology is a complete graph: the best individual among all islands in a given iteration is chosen to migrate to all islands. This does not maintain any diversity at migration points and is potentially dangerous when migration occurs frequently. We therefore wondered whether a sparser migration topology might be more beneficial with frequent migration. In an experiment, we have replaced the complete topology with a directed ring: during migration, each island selects the best individual among its current solution, and the current solution of its predecessor in the ring. Figure 2 displays the results in the setting with $n=75, t_0 = n^3$ and the directed ring topology used with $\tau = 1$, compared to the complete topology and $\tau = t_0$. Experimentally, choosing $\tau = 1, \lambda > 2$ and the ring migration topology appears to yield better probability of tracking Maze than $\tau = t_0, \lambda = 5$ and the complete migration topology.

## Conclusions

We have presented a first runtime analysis of parallel EAs in dynamic optimization. A simple island model with $\lambda$ islands and length of the migration interval $\tau$ was studied on the dynamic benchmark function Maze. In the case of extreme communication, i. e., $\tau =1$, even a large number of islands does not allow efficient tracking of the optimum. However, with a carefully chosen value for $\tau$, already a logarithmic number of islands was proven to be sufficient for efficient tracking. Finally, the relationship of $\tau , \lambda$, and the ability of the island model to track the optimum was investigated more closely. Our results indicate that the careful choice of the migration policy, and more generally, communication in parallel EAs, can be more advantageous than a large offspring population. Negative results have also been rigorously proved, showing that the island model will lose track of the optimum if it is not parameterized carefully.

Although most positive results heavily depend on a careful parametrization, our island model is less sensitive to other properties of Maze than previously considered approaches. In previous work, several approaches to optimizing Maze have been considered, but they all have significant limitations: MMAS, an ant colony algorithm, requires that the OPT individual is favored more often than the ALT individual in the oscillating pattern; a $(\mu +1)$ EA with genotype diversity exploits that the optimum oscillates between very few individuals, but requires both genotype diversity and an appropriate choice of $\mu$. The island model we consider requires careful choice of when migration is allowed to occur, but can, without additional changes, track the Maze if OPT is favored less often than ALT, or if Maze is extended to a finite-alphabet version.

In future work, the impact of the migration topology, something that we have only investigated experimentally here, could be analysed rigorously. Our initial results in this direction [18] rigorously prove, using a simplified model of Maze and the (1+1) islands, that there is a benefit to using a less-dense migration topology. The considered simplified model also relaxes the deterministic behaviour of the Maze oscillation, bringing it closer to modelling a noisy fitness function.

Additionally, we would like to study parallel EAs on different dynamic optimization problems in order to understand the interplay of migration intervals and number of islands more thoroughly. As our positive results are crucially dependent on a proper choice of $\tau$, it may also be worth studying adaptive or even self-adaptive choices of the migration interval in order to automatically determine a good value for $\tau$. Here the adaptive model suggested in [20] is of interest.
